# Love thy neighbour or opposites attract? Patterns of spatial segregation and association among crested penguin populations during winter

**DOI:** 10.1111/jbi.12279

**Published:** 2014-02-05

**Authors:** Norman Ratcliffe, Sarah Crofts, Ruth Brown, Alastair M M Baylis, Stacey Adlard, Catharine Horswill, Hugh Venables, Phil Taylor, Philip N Trathan, Iain J Staniland

**Affiliations:** 1 British Antarctic SurveyHigh Cross, Cambridge, CB3 0ET, UK; 2 Falklands ConservationJubilee Villas, Stanley, FIQQ 1ZZ, Falkland Islands; 3 Birdlife InternationalWellbrook Court, Cambridge, CB3 0NA, UK

**Keywords:** *Eudyptes*, Falkland Islands, geolocation, habitat selection, niche partitioning, penguin, South Georgia, spatial segregation, winter distribution

## Abstract

**Aim:**

Competition for food among populations of closely related species and conspecifics that occur in both sympatry and parapatry can be reduced by interspecific and intraspecific spatial segregation. According to predictions of niche partitioning, segregation is expected to occur at habitat boundaries among congeners and within habitats among conspecifics, while negative relationships in the density of species or populations will occur in areas of overlap. We tested these predictions by modelling the winter distributions of two crested penguin species from three colonies in the south-western Atlantic.

**Location:**

Penguins were tracked from two large colonies on the Falkland Islands and one in South Georgia, from where they dispersed through the South Atlantic, Southern Ocean and south-eastern Pacific.

**Methods:**

Forty macaroni penguins (*Eudyptes chrysolophus*) from South Georgia and 82 southern rockhopper penguins (*Eudyptes chrysocome chrysocome*) from two colonies in the Falkland Islands were equipped with global location sensors which log time and light, allowing positions to be estimated twice-daily, from April to August in 2011. Positions were gridded and converted into maps of penguin density. Metrics of overlap were calculated and density was related to remote-sensed oceanographic variables and competitor density using generalized additive models.

**Results:**

Macaroni penguins from western South Georgia and southern rockhopper penguins from Steeple Jason Island, Falkland Islands, were spatially segregated by differences in their habitat preferences thus supporting our first prediction regarding interspecific segregation. However, southern rockhopper penguins from Beauchêne Island showed a marked spatial overlap with macaroni penguins as the two had similar habitat preferences and strong mutual associations when controlling for habitat. Contrary to our predictions relating to intraspecific segregation, southern rockhopper penguins from Beauchêne Island and Steeple Jason Island were segregated by differences in habitat selection.

**Main conclusions:**

Morphological differentiation probably allows macaroni penguins from South Georgia and southern rockhopper penguins from Beauchêne Island to coexist in areas of spatial overlap, whereas segregation of the two Falkland rockhopper penguin populations may have arisen from two distinct lineages retaining cultural fidelity to ancestral wintering areas.

## Introduction

Competition for food has important influences upon animal foraging ecology, population regulation, community structure and speciation (Hutchinson, 1957; MacArthur & Levins, 1967; MacArthur, 1968). Interspecific competition occurs where two species compete for the same limited resource, with the result that the inferior competitor either becomes extinct or undergoes a behavioural or evolutionary shift to a different niche (Gause, 1934; Hardin, 1960). Such shifts can occur along multiple axes of the niche hypervolume (*sensu* Hutchinson, 1957), including that of spatial segregation, in which niche partitioning occurs via vertical or horizontal displacement (MacArthur, 1958; Amarasekare, 2003). Such segregation is often underpinned by divergence into spatially structured habitat types and so species distributions are separated by habitat boundaries (Arlettaz, 1999; Lombarte *et al*., 2000).

Intraspecific competition may also be reduced by spatial segregation among parapatric populations or colonies of central-place foragers, in which density-dependent competition causes animals from different localities to occupy spatially discrete home-ranges when foraging (Wakefield *et al*., 2013). Because parapatric populations of the same species ought to occupy the same niche, their spatial segregation would be expected to occur via mutual avoidance within habitat types rather than at habitat boundaries (Thiebot *et al*., 2011a, 2013). The interplay of interspecific and intraspecific competition is likely to be complex where conspecifics and congeners occur in sympatric and parapatric colonies: few studies have convincingly quantified spatial segregation in these circumstances or the role that habitat preference plays in this (Wakefield *et al*., 2011).

Macaroni penguins, *Eudyptes chrysolophus* Brandt, 1837, and southern rockhopper penguins, *E. chrysocome chrysocome* J.R. Forster, 1781, are crested penguins that occupy broadly similar ecological niches. They are important consumers of marine resources (Boyd, 2002), feeding on swarming crustaceans and mesopelagic fish in offshore waters (Clausen & Pütz, 2002; Waluda *et al*., 2012). Within the south-western Atlantic, their breeding distributions are spatially segregated in relation to water masses (see Appendix S1 in Supporting Information), with macaroni penguins occurring mostly on islands south of the Polar Front and southern rockhopper penguins on islands in the Subtropical Zone. Their breeding distributions overlap slightly on islands off South America and the Falkland Islands where the two species occasionally hybridize (White & Clausen, 2002).

The apparent spatial segregation of macaroni and southern rockhopper penguin foraging habitats in the SW Atlantic during the breeding season is likely to be exaggerated by the locations of breeding islands and the limited foraging ranges of birds from these (Barlow & Croxall, 2002; Masello *et al*., 2010; Ludynia *et al*., 2013). In the Indian Ocean macaroni and eastern rockhopper penguins (*E. filholi*) breed sympatrically in large numbers on islands in the Subantarctic Zone (e.g. Crawford *et al*., 2003), but in the SW Atlantic no islands occur in this water mass, so the scope for overlap in breeding range is reduced. Foraging niche overlap is therefore better examined during the winter, when birds are free from central-place constraints for several months, allowing them to range over thousands of kilometres to access their preferred feeding habitats (Bost *et al*., 2009). Nonetheless, Thiebot *et al*. (2011b) found that while the geographical ranges of penguins expand during the winter compared to the breeding season, their habitat preferences remained similar across these seasonal stages, such that habitat-dependent patterns of interspecific segregation observed during the breeding period should be preserved to some degree during the winter.

Accordingly, we predict that (1) winter segregation among southern rockhopper and macaroni penguins would arise from differences in habitat preference evident during the breeding period and that any overlap would occur in subantarctic waters. Where such overlap occurs we predict (2) a negative correlation in their densities owing to interspecific competition. With regard to intraspecific competition we predict that (3) conspecifics from different colonies within an archipelago will intermingle.

We test these three predictions by examining the winter distribution and habitat preference of southern rockhopper penguins from the two largest colonies in the Falkland Islands and macaroni penguins from the largest breeding aggregation in South Georgia, using a combination of tracking data and remote-sensed habitat variables collected during the same year. This paper builds upon similar studies for the Indian Ocean by providing comparative findings from a novel oceanic environment and by modelling interpopulation association or avoidance independently of habitat preference.

## Materials and methods

### Tag deployments

Adult penguins were tracked in 2011 using geolocation sensors (GLS; Mk18H, British Antarctic Survey, Cambridge, UK) that log light and saltwater immersion but not sea-surface temperature (SST). Tags weighed 2 g (linear dimensions: 15 × 9 × 5 mm) and were attached to the birds using leg rings. Birds were captured as pairs at their nest and were sexed according to bill length and depth (measured to the nearest 0.1 mm using a Vernier calliper); males have larger bills than females. Deployments were timed to coincide with the moult period (March 2011) and recoveries with the birds’ return to the breeding colonies in spring (November 2011): both members of the pair attend their nest site at these times, there are no nest contents to disturb and the duration of the deployments is minimized. GLS tags were deployed on 40 macaroni penguins on Bird Island, South Georgia (54°01′ S 38°03′ W) and on 82 southern rockhopper penguins on the Falklands, which were divided equally between Steeple Jason Island (51°01′ S 61°23′ W) and the Beauchêne Island (52°92′ S, 59°21′ W; see Appendix S1 for maps of locations). The colonies are all regionally important: Bird Island and the immediately adjacent Willis Islands host 408,000 pairs of macaroni penguins representing 43% of the South Georgia population (Trathan *et al*., 2012) while Steeple Jason and Beauchêne host 121,400 and 105,800 pairs of southern rockhopper penguins, representing 38% and 33% of the Falklands population, respectively (Baylis *et al*., 2013). Steeple Jason Island and Beauchêne Island are 250 km apart and Bird Island is 1500 km to the east of these. Abbreviations used subsequently are SRP and BRP for southern rockhopper penguins from Steeple Jason Island and Beauchêne Island, respectively, and SMP for macaroni penguins from western South Georgia (Bird Island and Willis Islands).

### Phenology

*Eudyptes* penguins remain ashore for several weeks while they moult in the autumn, stay at sea for the entire winter period and then stay ashore for several weeks in spring as they establish territories prior to breeding (Bost *et al*., 2009). Therefore the start and end dates of the winter period were clearly demarcated by the immersion records of the GLS loggers.

### Estimation of positions and density

Twice-daily macaroni and southern rockhopper penguin locations from April to August were estimated by geolocation using the R package tripEstimation (Sumner *et al*., 2009; Thiebot & Pinaud, 2010). Movement parameters within the model were constrained by a speed of 3 km h^−1^ with an SD of 1.8, derived from a satellite tracking study of wintering southern rockhopper penguins in the SW Atlantic (Raya Rey *et al*., 2007). Positions were constrained to occur in open water using a land mask [derived from the General Bathymetric Chart of the Oceans (GEBCO) world coast map, http://www.gebco.net/] to prevent positions occurring on land and a monthly sea-ice mask (average monthly extents based on data from MyOcean, http://www.myocean.eu.org/) to prevent positions falling in areas with greater than 10% coverage of sea ice. Light data before the end of the period affected by the spring equinox (24 April), and after that affected by the autumn equinox (20 August), were discarded owing to latitude estimation proving unreliable during these periods when based on light data alone.

TripEstimation uses a Bayesian Markov chain Monte Carlo model to estimate positions with uncertainty. Five chains of 1000 iterations were simulated after an initial burn-in period of 500 iterations, which were discarded; plots of chains were overlaid to confirm model convergence. The most likely path that a penguin followed was derived from the posterior estimate of the time-series of primary locations (i.e. the estimated locations at the instant-in-time a dawn or dusk event occurred; Sumner *et al*., 2009). The tripEstimation outputs were validated by confirming that the tracks it produced were broadly comparable to outputs from BASTrak, the widely accepted method of obtaining position estimates from geolocation data (Phillips *et al*., 2004).

A map of time spent across a 0.2° resolution grid was derived by assigning the time difference between two sequential primary locations to the possible intermediate locations the bird might have visited during the time interval between them. These describe uncertainty arising from the precision of the two primary locations and the possible paths the birds followed between these, given their distances apart in time and space, and the travel speed of the animal (Sumner *et al*., 2009). The time spent at all of the possible intermediate locations (i.e. 5000 points for each pair of primary locations) for all birds was summed within the grid cells in which they occurred. Gridding the uncertainty in locations not only illustrates their precision but also provides some degree of smoothing compared to simple gridding of the most likely primary positions. This avoids the need for further smoothing by kernel density estimation and the making of assumptions inherent in that method (Sumner *et al*., 2009).

The time spent in each cell by all birds from a population was divided by the total time spent across the entire grid to produce a surface of the proportion of time spent by the population across the entire study area. The duration of deployments and number of fixes were the same among birds and so this approach does not result in bias due to unequal sampling of individuals. Finally, the cell proportions were multiplied by the number of breeding birds in each population to produce a surface of bird density. The tracking data presented here are freely and publicly available from the British Antarctic Survey Polar Data Centre (polardatacentre@bas.ac.uk).

### Overlap among species

Overlap was examined by delimiting the isopleths within which 50% and 95% of birds occurred for each population, which are the limits defined as encompassing the core and peripheral range, respectively, in previous studies (e.g. Kokubun *et al*., 2010). The areas of intersection of the isopleths were then extracted as polygons and mapped in ArcGIS 9.1 (ESRI, Redlands, CA, USA).

Two indices of overlap were derived from these areas of intersection. The percentage of population *i* that overlaps with population *j* was calculated from equation 1:


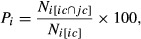
(1)

where *P* is the percentage, *c* is the contour of the 50% or 95% isopleth and *∩* is their area of overlap. An index of per-capita encounter rate of members of one population with another within the areas of intersection was calculated as the ratio (*R*) of *i*:*j* according to equation 2:


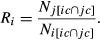
(2)

### Modelling habitat selection and interspecific associations

The proportion of birds from each population that occurred in each of the major water masses in the study area were extracted by clipping bird densities within water mass polygons (generated from sea surface height data; see below) in ArcGIS.

Density of birds from each population across the 0.2^o^ grid was modelled in relation to a suite of environmental covariates. The large-scale habitat variables used were depth (from GEBCO), dynamic height, which describes the locations of the major water masses and fronts (Venables *et al*., 2012, from AVISO, http://www.aviso.oceanobs.com), and chlorophyll *a* (chl-*a*) concentration (from NASA, http://oceancolor.gsfc.nasa.gov; see Appendix S1 for maps). Sea surface temperature (SST, from MyOcean) was also included in preliminary analyses but was correlated with dynamic height and so only the latter was used in models as it showed a lower degree of seasonal variability (Venables *et al*., 2012) and because the prey of penguins tend to be associated with specific water masses or fronts year-round rather than them switching water masses to track seasonal temperature changes (Ward *et al*., 2012). All variables were extracted and averaged within the time period over which penguin positions were estimated, except for chl-*a* data that were derived from a period between the 1 January to 15 April 2011 as an indication of the distribution of productivity prior to winter. Chl-*a* concentration data cannot be collected at high latitudes during mid-winter due to insufficient daylight to allow its estimation. The concentration of chl-*a* will be on average lower during winter than for the period that chl-*a* data were extracted but the relative distribution patterns will be broadly consistent. Cross-sea distance from the colony, derived using the ‘gridDistance’ function of the R package raster, was specified as a covariate to allow for the fact that not all cells were equally accessible from the source colony (Aarts *et al*., 2008).

Population group was included as a factor. In these models the intercept gives information on differences in average population density across the grid whereas the interactions with the smoothed habitat and competitor terms provide estimates of differences in habitat preference among groups. This approach is preferable to the more usual method of modelling each group separately as it allows direct statistical comparison of models in which groups do or do not differ in their habitat preferences: something that can only be done qualitatively when groups are modelled separately. Modelling all groups simultaneously also facilitates graphical comparisons of habitat preference as the partial residual plots of density versus habitat are derived from the same model rather than three different ones.

The degree to which a given population associated with or avoided the other two was modelled by including the density of each of the other populations as covariates. To avoid modelling the density of a population against itself, the covariates were intentionally aliaised where the population and competitor variables were the same (e.g. if the response variable was SMP density, the covariate describing SMP density was set to zero).

The habitat preference models were implemented using generalized additive models (GAMs) implemented within the R package mgcv (Wood, 2006). Models were fitted using the ‘bam’ rather than the ‘gam’ function of mgcv owing to the large size of the dataset and complexity of the model demanding a large allocation of memory. GAMs use nonparametric smoothers to fit flexible curves to data and so are suited to investigating the typically nonlinear relationships between animal densities and habitat variables (Aarts *et al*., 2008). Bird density was log+1 transformed to prevent negative predictions, and models were fitted with normal errors and an identity link, with their fit being checked by inspection of residual plots. The global model was specified with population group as a factor and for each covariate smooth terms were fitted within each group, using cubic shrinkage to identify the most parsimonious number of knots (Wood, 2006). Models were simplified by first removing the interactions of covariates with population group (so all populations had different intercepts but similar habitat preference according to that variable) and if these models were supported by removing the variable altogether (i.e. populations had different intercepts but showed no relationship with the given habitat variable).

Model selection was performed using the Akaike information criterion (AIC). Inspection of a variogram produced in the R package gstat (Pebesma, 2004) revealed spatial autocorrelation in the residuals of the global model, which invalidates the assumption of independence of data points inherent in AIC-based model selection. However, fitting a latitude–longitude tensor smooth with a 4° × 4° grid as a basis (Wood, 2006) removed spatial autocorrelation yet did not alter final model selection compared to the simpler model. The tensor smooth had the undesirable property of warping or flattening the biological relationships between density and explanatory variables owing to correlation (particularly dynamic height with latitude and depth and chl-*a* with longitude). We therefore present the AIC tables from models both with and without the tensor smooth but only present smoothed partial residual plots from the model without it.

## Results

### Device recoveries

Recovery rates of devices were high at 103 (79%) across all populations, 32 (80%) for SMP, 32 (78%) for SRP, and 34 (83%) for BRP. Data were downloaded successfully from all tags, but the batteries from one tag deployed on Bird Island expired in July and the data were discarded to maintain constancy in study period across all deployments.

### Phenology

The average colony departure dates after completion of moult were 13 April (SD = 3.5 days) for SMP, 17 April (SD = 3.0) for SRP and 26 March (SD = 3.9) for BRP. Return dates to the colonies were sex-specific, being earlier for males than females in all cases. Male SMP returned to Bird Island on 27 October (SD = 3.6) and females 8 days later (SD = 4.5). Male SRP arrived on 16 October (SD = 3.3) and females 3 days later (SD = 2.7) while male BRP returned on 5 October (SD = 4.1) and females 6 days later (SD = 3.1). The winter period therefore varied in duration from 182 to 205 days depending on site, sex and species. The number of days of the winter period (defined as the time between departure from the colony in autumn and the return to it in spring for the given population) for which positions could not be estimated ranged from 5 to 28 days during the spring equinox and from 52 to 76 days during the autumn one. The distribution patterns described below therefore only cover the central 35–42% of the wintering period.

### Winter distribution of density

**Figure 1:**
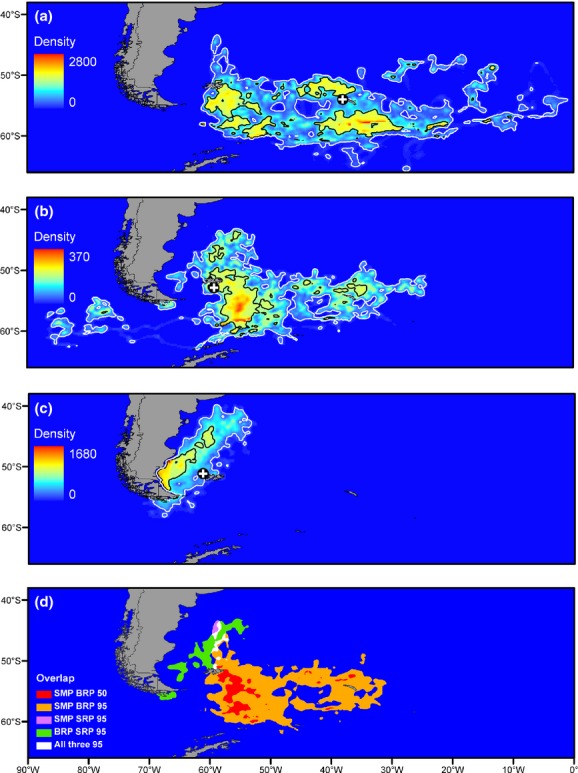
Distribution during the central wintering period of: (a) macaroni penguins (*Eudyptes chrysolophus*) from South Georgia (SMP); and southern rockhopper penguins (*E. chrysocome chrysocome*) from (b) Beauchêne Island (BRP) and (c) Steeple Jason Island (SRP). The black and white contour lines represent the 50% and 95% isopleths, respectively, and the black circle with white cross centre show the locations of the source colonies. Overlaps in the outer limits of the isopleths of the three populations are shown in panel (d).

The three populations showed marked differences in their distribution (Fig. 1; see Appendix S1 for locations of features mentioned). SMP had the widest distribution, ranging from 0 to 60° W and 43° to 66° S, although most occurred between 30° to 60° W and 51° to 62° S. Areas of elevated density were evident in the vicinity of fronts, particularly the Polar Front (PF) and Southern Antarctic Circumpolar Current Front (SACCF) to the south of South Georgia, the loop of the SACCF to the north of South Georgia and the Subantarctic Front, Patagonian Shelf and Burdwood Bank to the south of the Falkland Islands. Low densities were apparent in the large area of limited frontal activity to the west of South Georgia. BRP also ranged widely from 22° to 85° W and 43° to 62° S. However, most birds occurred within a relatively small area from 55° to 59° W and 50° to 59° S, with discrete patches of lower density around South Georgia and in the Pacific Ocean. SRP had a relatively restricted distribution, ranging from 55° to 70° W and 39° to 57° S. Density was highest in the relatively inshore waters in the Bahai Grande, particularly during the first half of the study period, after which some birds moved north-north-east towards the Patagonian Shelf break and Subantarctic Front (SAF). Individual movements followed a pattern of dispersive migration (*sensu* Newton, 2008) with birds radiating out from their colonies in various directions within their preferred habitats. No patterns of movement according to sex were evident for any population.

### Spatial overlap among populations

The three populations showed marked variation in their degree of spatial overlap (Fig. 1). The overlap of SRP and SMP was low: there was no overlap in core range and within the intersection of their peripheral ranges *P*_*i*_ was 0.8% for SMP ∩ SRP and 3.8% for SRP ∩ SMP. The overlap between SMP and BRP was substantial: within the intersection of their core ranges values of *P*_*i*_ were 22.9% for SMP ∩ BRP and 43.2% for BRP ∩ SMP while within that of their peripheral ranges values were 53.3% and 75.3%, respectively. The core ranges of the two SRP populations also did not overlap, and within the intersection of their peripheral ranges values of *P*_*i*_ were 8.2% for BRP ∩ SRP and 22.4% for SRP ∩ BRP. Within the area where the peripheral ranges of all three populations intersected, P_*i*_ was 0.5% for SMP, 2.1% for BRP and 2.6% for SRP.

*R*_*i*_ for SRP:SMP within their peripheral overlap was 0.5, such that SMP there were outnumbered 2:1.Values of *R*_*i*_ for SMP:BRP were 0.37 within their core overlap and 0.28 in their peripheral overlap, while that for SRP:BRP in their peripheral overlap was 0.26. Hence, BRP were outnumbered by approximately 3:1 within their areas of overlap with the other two populations.

### Habitat preference and interspecific relationships

The percentages of the three penguin populations found in each of the water masses are shown in Table 1. SMP occurred mainly in the Polar Frontal Zone (PFZ), Southern Antarctic Circumpolar Current Zone (SACCZ) and the Weddell Sea, with relatively few in the Subantarctic Zone (SAZ) and Subtropical Zone (STZ). SRP occurred mainly in the STZ, with smaller numbers in the SAZ and none in the more southerly water masses. BRP were intermediate, with most occurring in the SAZ and PFZ and fewer in the water masses to the north and south of these.

**Table 1:** The percentage of macaroni (*Eudyptes chrysolophus*) and southern rockhopper (*E. chrysocome chrysocome*) penguin populations that occur in the different water masses during winter. SMP denotes macaroni penguins from western South Georgia (Bird and Willis Islands) and SRP and BRP denote southern rockhopper penguins from Steeple Jason Island and Beauchêne Island, respectively. Water mass acronyms: STZ, Subtropical Zone; SAZ, Subantarctic Zone; PFZ, Polar Frontal Zone; SACCZ, Southern Antarctic Circumpolar Current Zone; WS, Weddell Sea

Water mass	SMP	BRP	SRP
STZ	9.5	17.7	85.8
SAZ	16.8	40.0	14.2
PFZ	29.9	31.5	0.0
SACCZ	22.3	10.1	0.0
WS	21.4	0.7	0.0

In the habitat modelling using GAMs, the model selection process showed that the global model received overwhelming support over reduced models based on AIC (Table 2), indicating that each of the populations showed differences in preference for each habitat variable and moreover that they exhibited association or avoidance with one another independently of habitat. The patterns of habitat selection and interspecific association or avoidance are shown in Appendix S2. The density of all populations tended to decline with distance from the breeding colony but then levelled off, indicating that habitat accessibility is an important factor affecting distribution, even during winter, despite the relaxation of central place constraints compared to the breeding season. SMP preferred relatively deep waters with lower chl-*a* concentration. Their relationship with dynamic height was striking, with a preference for the cold water mass of the Southern Antarctic Circumpolar Current Zone (SACCZ), but also peaks that coincide with the major fronts that separate the water masses. SRP showed preference for shallow waters that had high chl-*a* concentrations with high dynamic heights characteristic of subtropical and subantarctic waters. BRP selected waters of intermediate depth and ranges of dynamic height found in the Subantarctic and Polar Frontal Zones that were lower in chl-*a* than those preferred by SRP but higher than for SMP. There was also evidence of association and avoidance among the populations independently of fitted habitat variables. BRP and SMP density showed a strong positive relationship, whilst weaker and generally negative relationships were evident among these two populations and SRP.

**Table 2:** Selection among habitat distribution models for macaroni (*Eudyptes chrysolophus*) and southern rockhopper (*E. chrysocome chrysocome*) penguin density in the south-western Atlantic according to the Akaike information criterion (AIC). SMP denotes macaroni penguins from western South Georgia (Bird and Willis Islands) and SRP and BRP denote southern rockhopper penguins from Steeple Jason and Beauchêne Islands, respectively. The letter ‘s’ before a variable indicates a smooth term. The global model is that containing the interaction between the population variable and each of the habitat [distance from colony, depth, sea surface height, chlorophyll *a* (chl-*a*) concentration] and competitor terms (SMP, BRP and SRP), while other model names indicate the interaction term that was removed from the global model. AIC values are shown for the group of models with the tensor smooth fitted to allow for spatial autocorrelation of points and without it: note that the global model receives overwhelming support in both instances

Model	d.f.	AIC	ΔAIC
With tensor smooth			
Global	586.7	258226.1	0.0
Population × s(SMP)	586.2	258525.9	299.8
Population × s(SRP)	577.5	258609.4	383.3
Population × s(depth)	573.7	258825.3	599.2
Population × s(BRP)	584.8	259387.4	1161.3
Population × s(chl-*a*)	576.4	259420.3	1194.2
Population × s(dynamic height)	572.4	260763.3	2537.2
Population × s(distance)	574.2	261324.0	3097.9
Without tensor smooth			
Global	155.2	413044.4	0.0
Population × s(SMP)	146.5	413898.6	854.2
Population × s(SRP)	139.7	414811.7	1767.3
Population × s(depth)	137.3	416475.0	3430.6
Population × s(BRP)	139.5	419103.9	6059.5
Population × s(distance)	148.3	419117.5	6073.1
Population × s(chl-*a*)	137.3	422777.2	9732.8
Population × s(dynamic height)	147.2	426008.5	12964.1

## Discussion

### Winter distribution

Our data are the first to describe the winter distribution of macaroni penguins from South Georgia. Prior expectations were for birds to winter along the Polar Front to the north of South Georgia as they generally forage at this feature during their long incubation and pre-moult trips (Barlow & Croxall, 2002; Waluda *et al*., 2010). The birds did indeed use this area, but also dispersed far more widely over the Scotia Sea than previously believed, with unexpected aggregations to the south of the Falkland Islands, to the south-east of South Georgia and to the north-east of Elephant Island. The density and range of macaroni penguins around the Falklands in our study were far greater than those detected during winter at-sea surveys, probably due to half of the *Eudyptes* penguins seen being identified only to the genus level, all of which were later assumed to be southern rockhopper penguins (White *et al*., 2002).

The distribution of SRP found in our study was very similar to that found for birds tracked with satellite tags from three other colonies in the Falklands, with birds mainly wintering close to the Argentine coast to the west of the Falklands and some birds moving north along the Patagonian Shelf to around 40° S (Pütz *et al*., 2002). Our tracks from BRP revealed a very different winter distribution to that of birds from other colonies in the Falklands, including unexpected concentrations well to the south of the islands and around South Georgia, and two birds moving through the Drake Passage into the Pacific Ocean. These colony-specific patterns were also evident from satellite tracking of four birds from each of our study colonies that were collected simultaneously with our GLS data (Falklands Conservation, unpublished data). The winter distribution of BRP was similar to that of satellite-tracked southern rockhopper penguins from Staten Island in southern Argentina (Raya Rey *et al*., 2007). They also wintered to the south of the Falkland Islands with some movements into the Pacific, although their centre of distribution lay to the west of the main concentration of BRP.

### Interspecific segregation

Competition for food has been shown to lead to spatial niche partitioning among closely related penguin species (Kokubun *et al*., 2010; Wilson, 2010). According to prediction (1) we expected that winter segregation among southern rockhopper and macaroni penguins would arise from differences in habitat preference evident during the breeding period and that any overlap would occur in subantarctic waters. Moreover, according to prediction (2) we expected their densities to be negatively correlated owing to competition where they did co-occur. These predictions were supported by the relative distributions of SMP and SRP, which exhibited very little overlap owing to the former preferring deep and cold oceanic waters of PFZ and SACCZ while the latter preferred shallow, warm and productive shelf waters of the STZ. They co-occurred mainly in the SAZ and to a lesser extent in the STZ, with no overlap in more southerly water masses. There was weak evidence of interspecific avoidance independent of habitat selection, suggesting that partitioning among the two species was mainly achieved through differences in their habitat preferences with a lesser role played by mutual avoidance within the same habitats.

BRP distribution contradicted predictions (1) and (2) as they showed a preference for colder and deeper waters of the SAZ and PFZ rather than those of the STZ found around their breeding colony, such that they overlapped considerably with SMP. Indeed, some individuals of the two species swapped their respective summer and winter distributions, with some SMP wintering near the Falkland Islands and some BRP around South Georgia. Even when controlling for habitat preferences, SMP and BRP showed a strong positive association rather than competitive avoidance, perhaps owing to both selecting patches of elevated food availability that were not spatially correlated with habitat variables in our model. Similarly, high spatial overlap in the winter distribution of macaroni and eastern rockhopper penguins has been observed at the Crozet and Kerguelen archipelagos in the Indian Ocean where the two species also breed sympatrically (Thiebot *et al*., 2011a, 2012).

High levels of interspecific competition would be expected in those areas where these ecologically similar congeners co-occur. However, macaroni penguins are 74% larger than southern rockhopper penguins and so are able to dive deeper and handle bigger prey (Pütz *et al*., 2001, 2006; Green *et al*., 2005; Waluda *et al*., 2012). Niche partitioning may therefore occur along these axes of the niche hypervolume, as also found for sympatrically breeding *Pygoscelis* penguin species (Kokubun *et al*., 2010; Wilson, 2010). In contrast, northern rockhopper penguins (*E. moseleyi*) in the Indian Ocean exhibited little spatio-temporal overlap with eastern rockhopper penguins (Thiebot *et al*., 2012), perhaps because their similar body sizes would result in overlapping dive depths and prey size such that high levels of interspecific competition would have arisen had they co-occurred.

### Intraspecific segregation

Spatial segregation may arise among conspecifics from neighbouring colonies when foraging from a central place owing to density-dependent competition (Wakefield *et al*., 2013). Support for this theory has been found for numerous studies of seabirds during the breeding season (Ainley *et al*., 2004; Catry *et al*., 2013; Wakefield *et al*., 2013), including macaroni and southern rockhopper penguins (Trathan *et al*., 2006; Masello *et al*., 2010). During winter, when central place constraints are removed and birds are able to travel for larger distances over a period of months, populations might be expected to intermingle in the most profitable habitats. However, emerging evidence shows that parapatric and allopatric breeding populations of some pelagic seabird species may segregate spatially even during the winter (Appendix S3). Of most relevance to our study, Thiebot *et al*. (2011a, 2012) found that the winter distribution of both macaroni and eastern rockhopper penguins from the Crozet and Kerguelen archipelagos (1400 km apart) showed high conspecific spatial segregation within habitat types, while birds from different colonies within archipelagos intermingled. We therefore predicted that (3) BRP and SRP, being from colonies in the same archipelago, would share common wintering areas. This prediction was not supported as we found striking evidence of conspecific spatial segregation despite the colonies being a mere 250 km apart: a distance that penguins could travel in 3.5 days of their 200-day-long winter period. Moreover, and also contrary to prediction (3), the segregation was achieved primarily by BRP and SRP occupying different habitat types rather than them occupying non-overlapping areas of the same habitat. Birds from the two colonies therefore behaved more like different species than parapatric populations of the same species. This is perhaps the most extreme example of parapatric segregation during winter for any pelagic seabird documented to date.

The avoidance of the most productive habitats over the Patagonian Shelf by BRP is counter-intuitive. First impressions suggest that competitive exclusion by SRP is responsible, with birds from that colony gaining an advantage by being slightly closer to the most productive habitats than those from Beauchêne Island. However, as BRP start winter migration 3 weeks earlier than SRP birds (see Results) they could easily reach these areas first such that exclusion would be in the opposite direction to that observed.

The fact that the timing of breeding, winter distribution and habitat use of southern rockhopper penguins from Beauchêne Island is more similar to those from Staten Island (Raya Rey *et al*., 2007) than to those from colonies elsewhere in the Falklands (Pütz *et al*., 2002) raises the possibility that the Falklands population comprises two distinct lineages. Steeple Jason, and probably the remainder of the Falkland Islands apart from Beauchêne, may have been colonized by a lineage of southern rockhopper penguins in the distant past, after which birds selected the most productive wintering areas on the Patagonian Shelf and adjusted their phenology to match that of local oceanic productivity. Southern rockhopper penguins on Beauchêne Island may have colonized more recently from South America and maintained a cultural fidelity to their ancestral wintering habitats and breeding phenology. Genetic isolation of the two lineages may be maintained by a combination of colony fidelity and allochrony that arises from differences in the migration ecology of the two populations, as found for two parapatric Cook's petrel (*Pterodroma cookii*) populations in New Zealand (Rayner *et al*., 2011). Similarly, Thiebot *et al*. (2013) propose that winter distributions of crested penguins from different colonies in the Indian Ocean arise from migration paths that were formed under palaeoceanographic conditions and have since been preserved by cultural fidelity. The phylogeny of southern rockhopper penguins from Beauchêne and Steeple Jason islands requires further investigation in order to test this hypothesis.

Our understanding of spatial segregation of crested penguins populations in the south Atlantic and south-eastern Pacific is incomplete owing to a lack of tracking data from other important colonies in the region (Appendix S1). GLS tracking data are currently being collected from colonies in Argentina, Chile and Tristan da Cunha (pers. comm. respectively with: A. Reya Rey, Consejo Nacional de Investigaciones Científicas y Técnicas; D. Oehler, Wildlife Conservation Society; and R. Cuthbert, RSPB) which will complete our knowledge of how southern and northern rockhopper penguins partition space during the winter. There are no winter tracking data of macaroni penguins from the South Sandwich Islands, and satellite images suggest that the population size there is likely to be far greater than previously thought (H. Lynch, Stony Brook University, pers. comm.). We hypothesize that the relatively low usage of eastern waters by South Georgia macaroni penguins (Fig. 1a) arises from competitive exclusion by conspecifics from the South Sandwich Islands. Testing this hypothesis will be logistically challenging owing to the extreme difficulties in accessing these remote and exposed islands.

## Biosketch

**Norman Ratcliffe** is a seabird ecologist working at British Antarctic Survey. His main research interests are in the foraging ecology and movements of seabirds in relation to environmental and biotic variables, and their repercussions upon individual fitness and population processes.

Author contributions: The concept of this study was developed by N.R. and P.N.T. Fieldwork was conducted by N.R., S.C., R.B., A.M.M.B., S.A. and C.H.; P.T. and I.J.S. contributed to modelling of the tracking data and H.V. to the sourcing and manipulation of the environmental variables. N.R. wrote the paper with contributions from all the other authors.

## SUPPORTING INFORMATION

**Appendix S1** Estimated populations (breeding pairs) of southern rockhopper and macaroni penguins in the study area, and maps of locations and status of important crested penguin breeding aggregations, the distribution of environmental covariates used for modelling and place names mentioned in the text.

**Appendix S2** Partial residual plots of the response of penguin density to linear predictors in the global model.

**Appendix S3** Published studies of winter-period spatial segregation among colonies of conspecific seabirds based on data from tracking devices.
