# Supplementary material for: Love thy neighbour or opposites attract? Patterns of spatial segregation and association among crested penguin populations during winter
Source: J Biogeogr. 2014 Feb 5;41(6):1183–92. doi: 10.1111/jbi.12279 (PMC4255236; doi:10.1111/jbi.12279)
Supplement: Supplementary file 1 — Appendix S1 Estimated populations (breeding pairs) of southern rockhopper and macaroni penguins in the study area, and maps of locations and status of important crested penguin breeding aggregations, the distribution of environmental covariates used for modelling and place names mentioned in the text. [file jbi0041-1183-sd1.doc]

*Journal of Biogeography*

**Supporting Information**

**Love thy neighbour or opposites attract? Patterns of spatial segregation and association among crested penguin populations during winter**

Norman Ratcliffe, Sarah Crofts, Ruth Brown, Alastair M. M. Baylis, Stacey Adlard, Catharine Horswill, Hugh Venables, Phil Taylor, Philip N. Trathan and Iain J. Staniland

**Appendix S1** Estimated populations (breeding pairs) of southern rockhopper and macaroni penguins in the study area, and maps of locations and status of important crested penguin breeding aggregations, the distribution of environmental covariates used for modelling and place names mentioned in the text.

**Appendix S1a** Estimated populations from the most recent censuses of important crested penguin colonies (> 5000 pairs) in the study area. ID numbers cross reference to the colony locations shown in Appendix S1b. RP is the number of breeding pairs of southern rockhopper penguins (*E. chrysocome chrysocome*); MP is the number of breeding pairs of macaroni penguins (*Eudyptes chrysolophus*). Acronyms for the water masses within which colonies occur are given in the footnote of Appendix S1b.

| ID | Colony name | RP | MP |  | Water  mass | Reference |
| --- | --- | --- | --- | --- | --- | --- |
| 1 | Isla Noir, Chile | 158,200 | 3,470 |  | STZ | Oehler *et al*. (2008) |
| 2 | Isla Ildefonso, Chile | 86,400 | 5,660 |  | STZ | Kirkwood *et al*. (2007) |
| 3 | Isla Diego Ramirez, Chile | 132,721 | 15,600 |  | STZ | Kirkwood *et al*. (2007) |
| 4 | Barnevelt, Chile, Chile | 7,000 | 50 |  | STZ | Bingham & Mejias (1999) |
| 5 | Staten Island, Argentina | 173,793 | 50 |  | STZ | Schiavini (2000);  Bingham & Mejias (1999) |
| 6 | Falkland Islands, UK | 319,163 | 24 |  | STZ | Baylis *et al*. (2013);  Huin (2007) |
| 7 | South Georgia, UK | 0 | 938,017 |  | PFZ | Trathan *et al*. (2012) |
| 8 | South Sandwich Islands, UK | 0 | 56,128 |  | SACCZ | Convey *et al*. (1999) |

**References**

Baylis, A.M.M., Wolfaardt, A.C., Crofts, S., Pistorius, P.A. & Ratcliffe, N. (2013) Increasing trend in the number of southern rockhopper penguins (*Eudyptes c. chrysocome*) breeding at the Falkland Islands. *Polar Biology*, **36**, 1007-1018.

Bingham, M. & Mejias, E. (1999) Penguins of the Magellan region. *Scientia Marina*, **63**, 485-493.

Convey, P., Morton, A. & Poncet, J. (1999) Survey of marine birds and mammals of the South Sandwich Islands. *Polar Record*, **35**, 107-124.

Huin, N. (2007) *Falklands Island penguin census*. Falklands Conservation, Stanley, Falkland Islands.

Kirkwood, R., Lawton, K., Moreno, C., Valencia, J., Schlatter, R. & Robertson, G. (2007) Estimates of southern rockhopper and macaroni penguin numbers at the Ildefonso and Diego Ramirez Archipelagos, Chile, using quadrat and distance-sampling techniques. *Waterbirds*, **30**, 259-267.

Oehler, D.A., Pelikan, S., Fry, W.R., Weakley, L., Jr, Kusch, A. & Marin, M. (2008) Status of crested penguin (*Eudyptes* spp.) populations on three islands in southern Chile. *Wilson Journal of Ornithology*, **120**, 575-581.

Schiavini, A.C.M. (2000) Staten Island, Tierra del Fuego: the largest breeding ground for southern rockhopper penguins? *Waterbirds*, **23**, 286-291.

Trathan, P.N., Ratcliffe, N. & Masden, E.A. (2012) Ecological drivers of change at South Georgia: the krill surplus, or climate variability? *Ecography*, **35**, 983-993.

**Appendix S1b** Maps of the study area showing (a) bathymetry (m depth), locations of important crested penguin breeding aggregations (black circles; white numbers cross reference to ID column in Appendix S1a), notable place names including those mentioned in the text (white numbers, black letters). Black boxes around points 6 and 7 are insets for the detailed maps shown in Appendix S1c. (b) dynamic height (cm) and the locations of major fronts and water masses and (c) chl-*a* concentration (mg/m^3^).


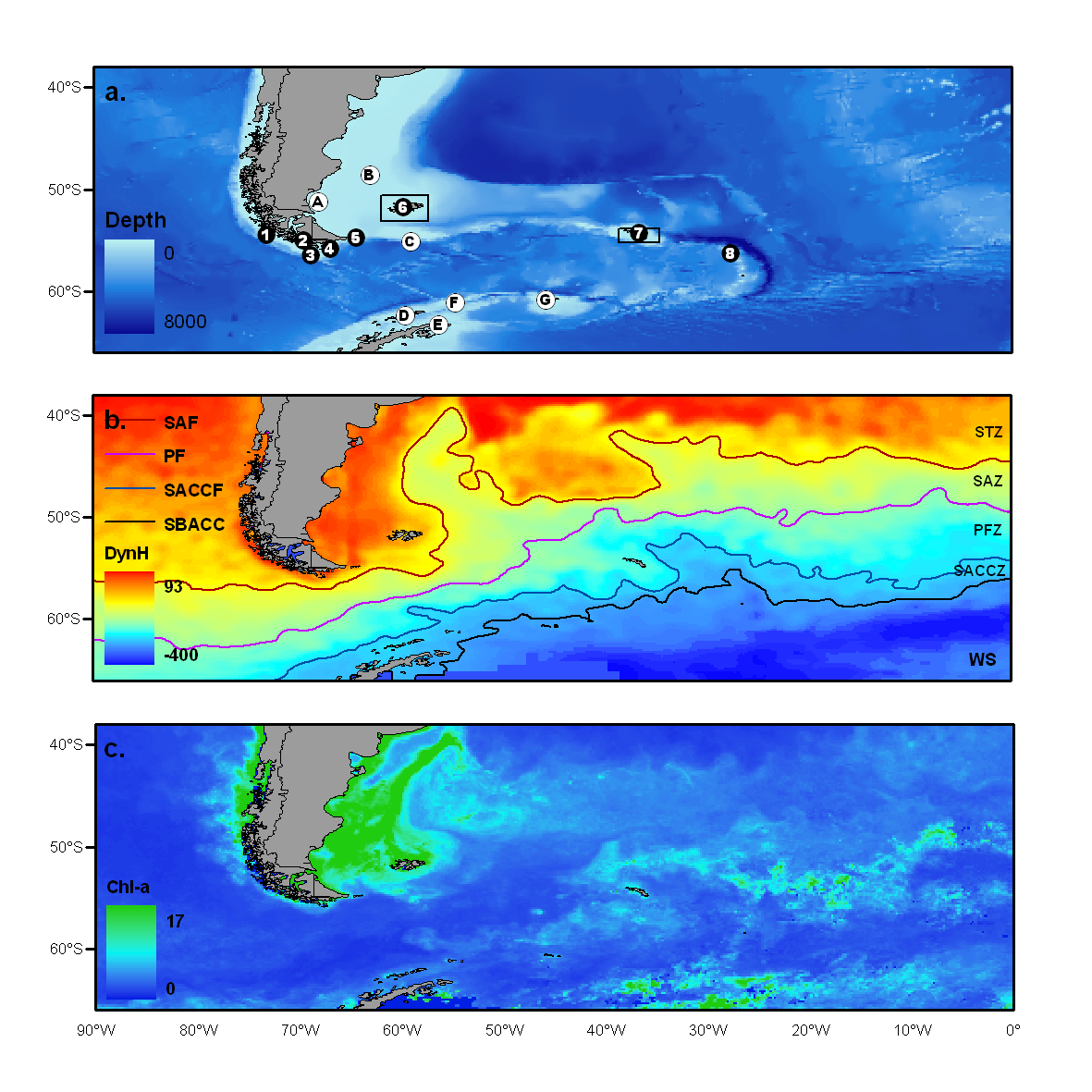


*Footnotes*: 1a. Place names: A, Bahia Grande; B, Patagonian Shelf; C, Burdwood Bank; D, South Shetland archipelago; E, Antarctic Peninsula; F, Elephant Island; G, South Orkney Islands. 1b. Front acronyms: SAF, Subantarctic Front; PF, Polar Front; SACCF, Southern Antarctic Circumpolar Current Front; SBACC, Southern Boundary of Antarctic Circumpolar Current. Water mass acronyms: STZ, Subtropical Zone; SAZ, Subantarctic Zone; PFZ, Polar Frontal Zone; SACCZ, Southern Antarctic Circumpolar Current Zone; WS, Weddell Sea.

**Appendix S1c** Locations and sizes of (i) southern rockhopper penguin colonies in the Falkland Islands (after Baylis *et al*., 2013) and (ii) macaroni penguins in South Georgia (after Trathan *et al*., 2012). Symbols indicate colony size, with blue showing the locations from which birds were tracked [SJI, Steeple Jason Island; BI, in (i) Beauchêne Island and in (ii) Bird Island] and yellow other colonies. Those colonies on the Falkland map marked with a black dot in the centre are those at which small numbers of macaroni penguins have also been recorded breeding (Huin *et al*., 2007).

**
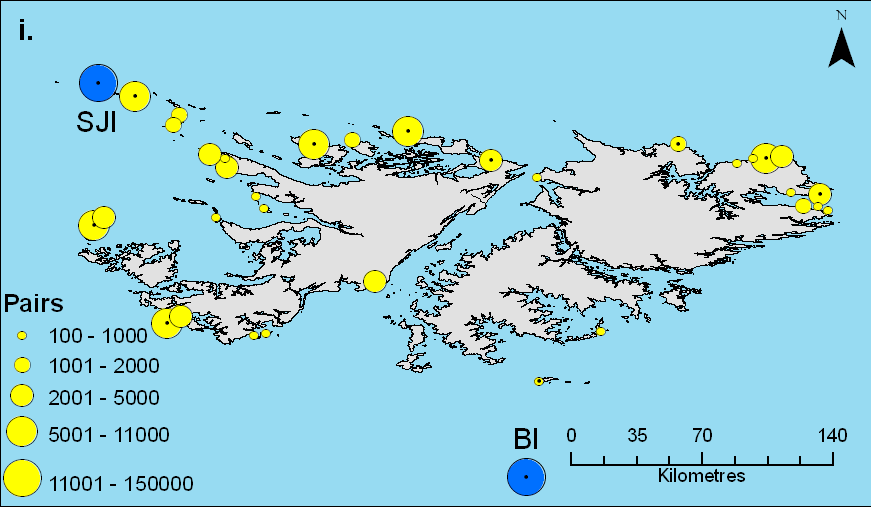
**

**
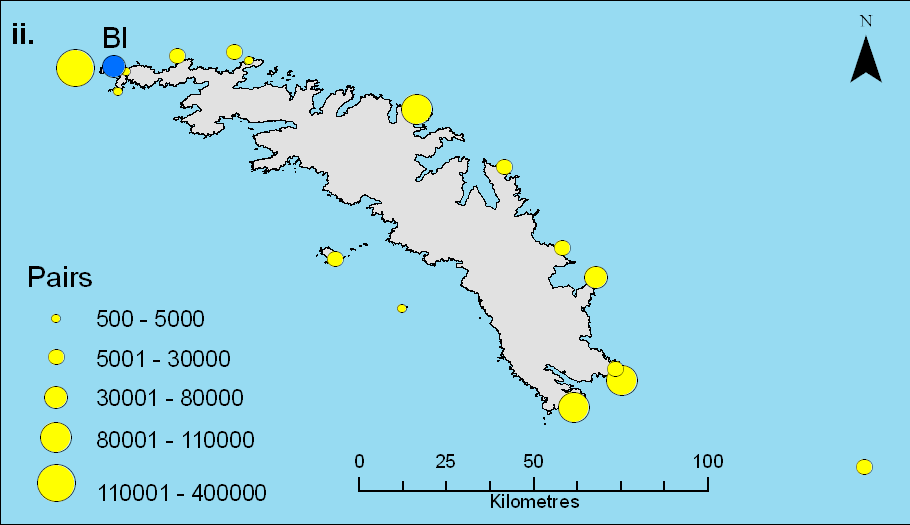
**
