# Supplementary material for: Love thy neighbour or opposites attract? Patterns of spatial segregation and association among crested penguin populations during winter
Source: J Biogeogr. 2014 Feb 5;41(6):1183–92. doi: 10.1111/jbi.12279 (PMC4255236; doi:10.1111/jbi.12279)

*Journal of Biogeography*

**Supporting Information**

**Love thy neighbour or opposites attract? Patterns of spatial segregation and association among crested penguin populations during winter**

Norman Ratcliffe, Sarah Crofts, Ruth Brown, Alastair M. M. Baylis, Stacey Adlard, Catharine Horswill, Hugh Venables, Phil Taylor, Philip N. Trathan

and Iain J. Staniland

**Appendix S2** Partial residual plots of the response of penguin density to linear predictors in the global model.

**Appendix S2a** Explanation of figures in Appendix S2b and S2c. The solid curves represent smoothed functions of the partial residuals when controlling for other variables included in the model, with the dotted curves flanking these representing the 95% confidence intervals. The rug plots along the *x*-axis denote values at which *y* data occur. Dotted vertical lines on the dynamic height figures show the values at which the major oceanic fronts occur: from left to right the Southern Boundary of the Antarctic Circumpolar Current, the Southern Antarctic Circumpolar Current Front, Polar Front and Subantarctic Front. Scales of the *y*-axis are fixed to allow magnitudes of effects to be compared across predictor variables. Population acronyms: SMP, western South Georgia macaroni penguin (*Eudyptes chrysolophus*); BRP, Beauchêne Island southern rockhopper penguin (*E. chrysocome chrysocome*); SRP, Steeple Jason Island southern rockhopper penguin. Note that plots is Appendix S2b and S2c are tabulated (columns are populations, rows are environmental variables) while in Appendix 2d each plot has its own unique *x*–*y* axis labels.

**Appendix S2b** Variation in density of macaroni and southern rockhopper penguins in the SW Atlantic in relation to distance from colony and depth.

**
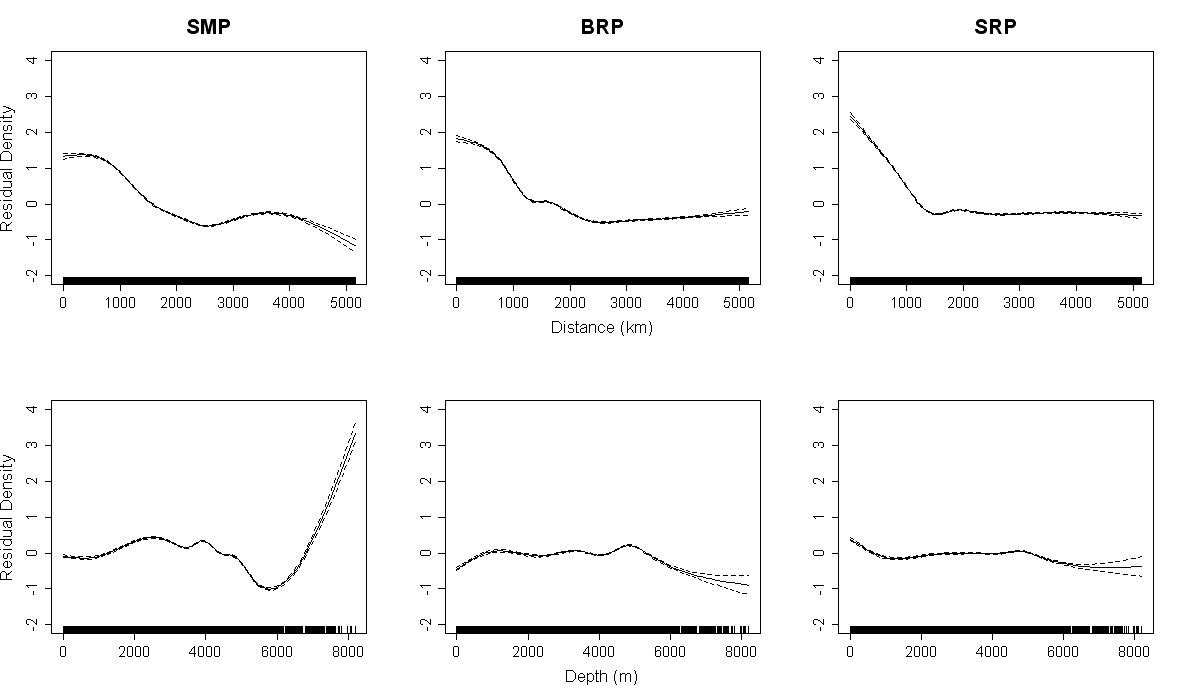
**

**Appendix S2c** Variation in density of macaroni and southern rockhopper penguins in the SW Atlantic in relation to dynamic height and chlorophyll *a* concentration.


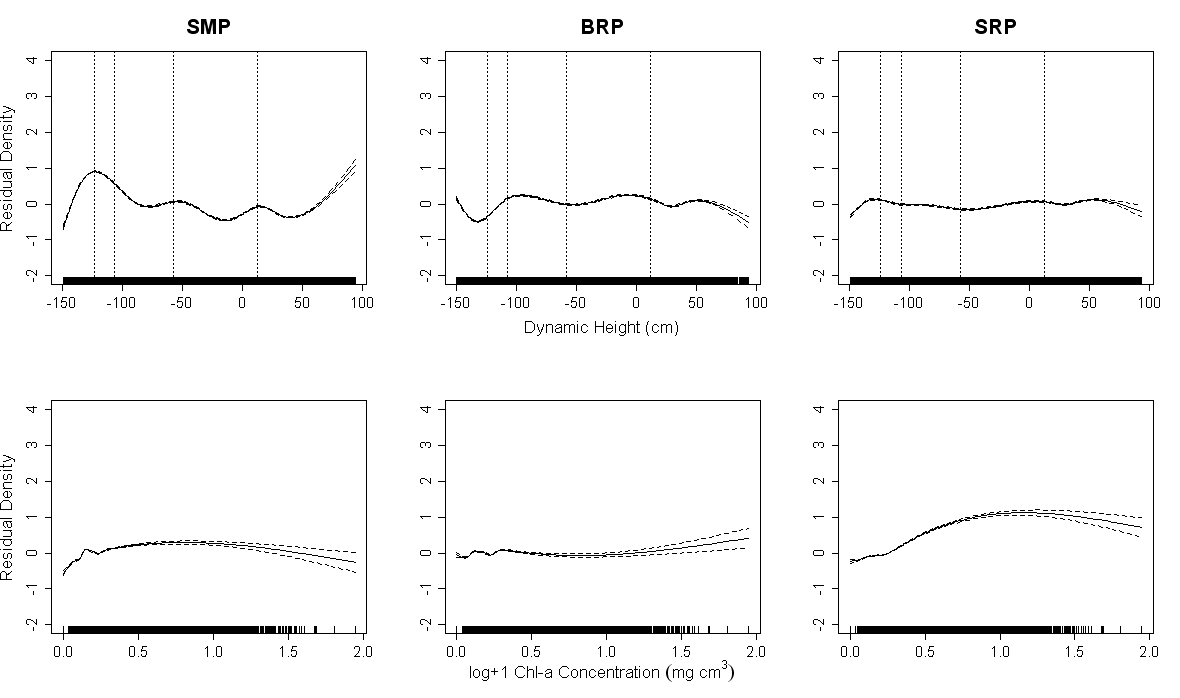


**Appendix S2d** Variation in density of macaroni and southern rockhopper penguins in the SW Atlantic in relation to competitor density.


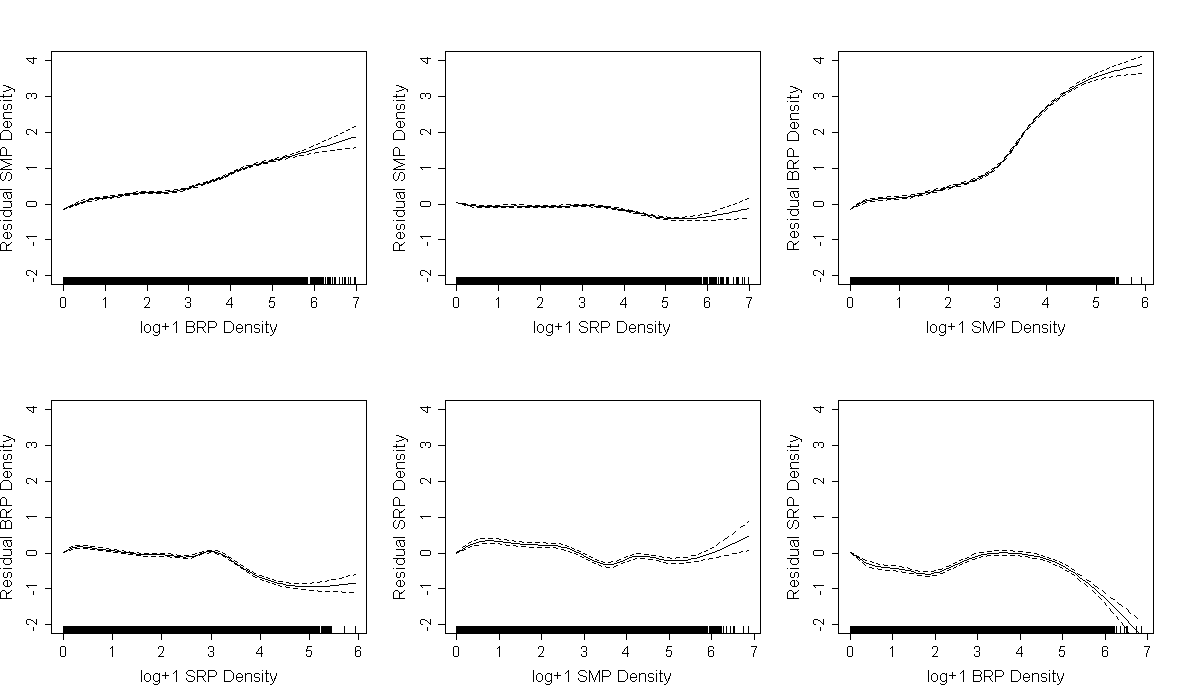

Supplement: Supplementary file 2 — Appendix S2 Partial residual plots of the response of penguin density to linear predictors in the global model. [file jbi0041-1183-sd2.doc]
