# Supplementary material for: Love thy neighbour or opposites attract? Patterns of spatial segregation and association among crested penguin populations during winter
Source: J Biogeogr. 2014 Feb 5;41(6):1183–92. doi: 10.1111/jbi.12279 (PMC4255236; doi:10.1111/jbi.12279)
Supplement: Supplementary file 3 — Appendix S3 Published studies of winter-period spatial segregation among colonies of conspecific seabirds based on data from tracking devices. [file jbi0041-1183-sd3.doc]

*Journal of Biogeography*

**Supporting Information**

**Love thy neighbour or opposites attract? Patterns of spatial segregation and association among crested penguin populations during winter**

Norman Ratcliffe, Sarah Crofts, Ruth Brown, Alastair M. M. Baylis, Stacey Adlard, Catharine Horswill, Hugh Venables, Phil Taylor, Philip N. Trathan and Iain J. Staniland

**Appendix S3** Published studies of spatial segregation among colonies of conspecific seabirds based on data from tracking devices. Device codes: PTT, platform terminal transmitter (satellite tag); GLS, global location sensor. Segregation scores: Complete = no overlap, High = some overlap of peripheral ranges, Moderate = overlap of peripheral ranges but no or slight overlap of core ranges, Low = considerable overlap of core ranges.

| Species | Device | Degree of segregation | Reference |
| --- | --- | --- | --- |
| *Eudyptes chrysolophus* | GLS | High | Thiebot *et al.* (2011, 2013) |
| *Eudyptes filholi* | GLS | High | Thiebot *et al.* (2012, 2013) |
| *Phoebastria immutabilis* | GLS | Moderate | Young *et al.* (2009) |
| *Fulmarus glacialis* | PTT | High through to low, depending on colonies compared | Hatch *et al.* (2010) |
| *Pterodroma cookii* | GLS | Low early and late winter but complete in central period | Rayner *et al.* (2011) |
| *Calonectris diomedea* | GLS | Low | González-Solís *et al.* (2007) |
| *Calonectris diomedea* | GLS | Low | Catry *et al.* (2011) |
| *Puffinus griseus* | GLS | Low | Shaffer *et al.* (2006) |
| *Morus bassanus* | GLS/PTT | Low | Montevecchi *et al.* (2012) and pers. comm. |
| *Catharacta skua* | GLS | High | Magnusdottir *et al.* (2011) |
| *Rissa tridactyla* | GLS | Low for most Atlantic colonies, but British ones show high segregation from others | Frederiksen *et al.* (2012) |
| *Sterna paradisaea* | GLS | Low although only one bird tracked from second colony | Egevang *et al.* (2010) |
| *Uria lomvia* | GLS | High through to low, depending on colonies compared | Gaston *et al.* (2011); McFarlane Tranquilla *et al.* (2013) |
| *Uria lomvia* | GLS | High to moderate, depending on colonies compared | Steen *et al*. (2013) |
| *Uria aalge* | GLS | Low | McFarlane Tranquilla *et al.* (2013) |

**References**

Catry, P., Dias, M.P., Phillips, R.A. & Granadeiro, J.P. (2011) Different means to the same end: Long-distance migrant seabirds from two colonies differ in behaviour, despite common wintering grounds. *PLoS ONE,* **6**, e26079.

Egevang, C., Stenhouse, I.J., Phillips, R.A., Petersen, A., Fox, J.W. & Silk, J.R.D. (2010) Tracking of Arctic terns *Sterna paradisaea* reveals longest animal migration. *Proceedings of the National Academy of Sciences USA,* **107**, 2078-2081.

Frederiksen, M., Moe, B., Daunt, F., Phillips, R.A., Barrett, R.T., Bogdanova, M.I., Boulinier, T., Chardine, J.W., Chastel, O., Chivers, L.S., Christensen-Dalsgaard, S., Clément-Chastel, C., Colhoun, K., Freeman, R., Gaston, A.J., González-Solís, J., Goutte, A., Grémillet, D., Guilford, T., Jensen, G.H., Krasnov, Y., Lorentsen, S.-H., Mallory, M.L., Newell, M., Olsen, B., Shaw, D., Steen, H., Strøm, H., Systad, G.H., Thórarinsson, T.L. & Anker-Nilssen, T. (2012) Multicolony tracking reveals the winter distribution of a pelagic seabird on an ocean basin scale. *Diversity and Distributions,* **18**, 530-542.

Gaston, A.J., Smith, P.A., McFarlane Tranquilla, L.A., Montevecchi, W.A., Fifield, D.A., Gilchrist, H.G., Hedd, A., Mallory, M.L., Robertson, G.J. & Phillips, R.A. (2011) Movements and wintering areas of breeding age Thick-billed Murre *Uria lomvia* from two colonies in Nunavut, Canada. *Marine Biology,* **158**, 1929-1941.

González-Solís, J., Croxall, J.P., Oro, D. & Ruiz, X. (2007) Trans-equatorial migration and mixing in the wintering areas of a pelagic seabird. *Frontiers in Ecology and the Environment,* **5**, 297-301.

Hatch, S.A., Gill, V.A. & Mulcahy, D.M. (2010) Individual and colony-specific wintering areas of Pacific northern fulmars (*Fulmarus glacialis*). *Canadian Journal of Fisheries and Aquatic Sciences,* **67**, 386-400.

Magnusdottir, E., Leat, E.H.K., Bourgeon, S., Strøm, H., Petersen, A., Phillips, R.A., Hanssen, S.A., Bustnes, J.O., Hersteinsson, P. & Furness, R.W. (2011) Wintering areas of Great Skuas *Stercorarius skua* breeding in Scotland, Iceland and Norway. *Bird Study,* **59**, 1-9.

McFarlane Tranquilla, L.A., Montevecchi, W.A., Hedd, A., Fitfield, D.A., Burke, C.M., Smith, P.A., Regular, P.M., Robertson, G.J., Gaston, A.J. & Phillips, R.A. (2013) Multiple-colony winter habitat use by murres *Uria* spp. in the Northwest Atlantic Ocean: implications for marine risk assessment. *Marine Ecology Progress Series,* **472**, 287-303.

Montevecchi, W., Fifield, D., Burke, C., Garthe, S., Hedd, A., Rail, J.-F. & Robertson, G. (2012) Tracking long-distance migration to assess marine pollution impact. *Biology Letters,* **8**, 218-221.

Rayner, M.J., Hauber, M.E., Steeves, T.E., Lawrence, H.A., Thompson, D.R., Sagar, P.M., Bury, S.J., Landers, T.J., Phillips, R.A., Ranjard, L. & Shaffer, S.A. (2011) Contemporary and historical separation of transequatorial migration between genetically distinct seabird populations. *Nature Communications,* **2**, article 332.

Shaffer, S.A., Tremblay, Y., Weimerskirch, H., Scott, D., Thompson, D.R., Sagar, P.M., Moller, H., Taylor, G.A., Foley, D.G., Block, B.A. & Costa, D.P. (2006) Migratory shearwaters integrate oceanic resources across the Pacific Ocean in an endless summer. *Proceedings of the National Academy of Sciences USA,* **103**, 12799-12802.

Steen, H., Lorentzen, E. & Strøm, H. (2013) *Winter distribution of guillemots (Uria spp.) in the Barents Sea*. Report Number 141, Norwegian Polar Institute, Tromsø, Norway.

Thiebot, J.-B., Cherel, Y., Crawford, R.J.M., Makhado, A.B., Trathan, P.N., Pinaud, D. & Bost, C-A. (2013) A space oddity: geographic and specific modulation of migration in *Eudyptes* penguins. *PLoS ONE*, **8**, e71429.

Thiebot, J.-B., Cherel, Y., Trathan, P.N. & Bost, C.-A. (2011) Inter-population segregation in the wintering areas of macaroni penguins. *Marine Ecology Progress Series,* **421**, 279-290.

Thiebot, J.-B., Cherel, Y., Trathan, P.N. & Bost, C.-A. (2012) Coexistence of oceanic predators on wintering areas explained by population-scale foraging segregation in space or time. *Ecology,* **93**, 122-130.

Young, L.C., Vanderlip, C., Duffy, D.C., Afanasyev, V. & Shaffer, S.A. (2009) Bringing home the trash: do colony-based differences in foraging distribution lead to increased plastic ingestion in Laysan albatrosses? *PLoS ONE,* **4**, e7623.
